# Should psychology be ‘positive’? Letting the philosophers speak: Commentary on... Hope, optimism and delusion

**DOI:** 10.1192/pb.bp.113.045823

**Published:** 2014-04

**Authors:** Femi Oyebode

**Affiliations:** 1 National Centre for Mental Health, Birmingham

## Abstract

This is a brief commentary on the value of optimism in therapy. It draws on the philosophical writings of Schopenhauer and Aristotle. It suggests that the modern preoccupation with optimism may be as extreme as the bleak pessimistic outlook favoured by Schopenhauer.

The nature of pessimism is best illustrated by the writings of Arthur Schopenhauer. In *On the Vanity of Existence*,^1^ he wrote:

’Yet what a difference there is between our beginning and our end! We begin in the madness of carnal desire and the transport of voluptuousness, we end in the dissolution of all our parts and the musty stench of corpses. And the road from one to the other too goes, in regard to our well-being and enjoyment of life, steadily downhill: happily dreaming childhood, exultant youth, toil-filled years of manhood, infirm and often wretched old age, the torment of the last illness and finally the throes of death - does it not look as if existence were an error the consequences of which gradually grow more and more manifest? We shall do our best to think of life as a *desengaño*, as a process of disillusionment: since this is, clearly enough, what everything that happens to us is calculated to produce.’ (p. 54)

For Schopenhauer, suffering is the inescapable condition of life. The true cure of the sickness of life is the acceptance of annihilation. With this bleak worldview in mind, the need in most human beings for hope and optimism becomes comprehensible. The reality of day-to-day life can be intolerable and given man’s capacity for abstract thought, for imagining absent and future things, fear and hope understandably arise out of this bleak perspective. Thus, living involves fear that the intolerable might continue and hope that things may change for the better. Hope in this context seems laudable.

In her editorial, Rebecca McGuire-Snieckus^2^ raises important questions about the role and purpose of therapies which derive from the assumption that ‘positive psychology’ is desirable, that is, therapies which trade on hope, on creating a positive belief about possible outcomes, even if falsely. Even though she does not explicitly raise the moral dimension to her critique - namely that even if positive psychology can be empirically shown to be beneficial, to make people happier for instance, is it morally right to instil hope in hopeless situations? - the questions that she raises go to the very heart of what healthcare is about. And there are no easy answers.

Norman Vincent Peale popularised the notion of ‘positive thinking’, the idea that people can change their lives by changing their thoughts. The implication is that personal problems and failure are manifestations of ‘negative thoughts’. If only people conscientiously applied Peale’s principles, they would be able to turn their lives around. In this regard immovable external objects obstructing personal progress exist only in the mind’s eye.

While Seligman^3^ draws a distinction between ‘positive thinking’ and ‘positive psychology’, the truth is that the same preoccupation is at play, namely that pessimism is bad for you, that your manner of thinking can make you happier, more successful, and that your thinking can be changed for the better.

Both polarities, in my view, seem unhelpful. Schopenhauer’s pessimism paints a bleak and foreboding picture of the world and of existence. Peale’s positive thinking and aspects of Seligman’s positive psychology unduly exaggerate the importance of happiness, at all costs, as a goal of existence.

I leave the last word to Aristotle, who wrote on ‘sanguineness or optimism’ as follows:^4^

‘The sanguine are confident because they think they are the best soldiers and cannot lose (this is how people behave when they get drunk: they become sanguine); but when the result does not turn out as expected, they run away... it is the mark of a courageous man to face things that are terrible to a human being, and that he can see as such, because it is a fine act to face them and a disgrace not to do so’.
